# Phosphatidylinositol 5-phosphate 4-kinase regulates early endosomal dynamics during clathrin-mediated endocytosis

**DOI:** 10.1242/jcs.202259

**Published:** 2017-07-01

**Authors:** Kumari Kamalesh, Deepti Trivedi, Sarah Toscano, Sanjeev Sharma, Sourav Kolay, Padinjat Raghu

**Affiliations:** 1 National Centre for Biological Sciences-TIFR, GKVK Campus, Bellary Road, Bangalore 560065, India; 2 Department of Biological Sciences, Tata Institute of Fundamental Research, Dr. Homi Bhabha Road, Colaba, Mumbai 400005, India; 3 Inositide Laboratory, Babraham Institute, Cambridge CB22 3AT, UK; 4 Manipal University, Madhav Nagar, Manipal, Karnataka 576104, India

**Keywords:** *Drosophila*, Endosome, Phosphoinositide kinase, Photoreceptor

## Abstract

Endocytic turnover is essential for the regulation of the protein composition and function of the plasma membrane, and thus affects the plasma membrane levels of many receptors. In *Drosophila melanogaster* photoreceptors, photon absorption by the G-protein-coupled receptor (GPCR) rhodopsin 1 (Rh1; also known as NinaE) triggers its endocytosis through clathrin-mediated endocytosis (CME). We find that CME of Rh1 is regulated by phosphatidylinositol 5 phosphate 4-kinase (PIP4K). Flies lacking PIP4K show mislocalization of Rh1 on expanded endomembranes within the cell body. This mislocalization of Rh1 was dependent on the formation of an expanded Rab5-positive compartment. The Rh1-trafficking defect in PIP4K-depleted cells could be suppressed by downregulating Rab5 function or by selectively reconstituting PIP4K in the PI3P-enriched early endosomal compartment of photoreceptors. We also found that loss of PIP4K was associated with increased CME and an enlarged Rab5-positive compartment in cultured *Drosophila* cells. Collectively, our findings define PIP4K as a novel regulator of early endosomal homeostasis during CME.

## Abstract (precis)

**Highlighted Article:** Phosphatidylinositol 5-phosphate 4-kinase regulates early endosomal dynamics and trafficking of plasma membrane receptors through clathrin-mediated endocytosis in *Drosophila* cells.

## INTRODUCTION

The ability to sense and respond to changes in local milieu is fundamental to survival. To achieve this, the plasma membrane (PM) of a cell has a defined protein composition, including the specific numbers and types of receptors required to respond to environmental stimuli. The control of composition requires not only transport events that deliver proteins to the PM but also tight control of endocytic processes that can remove proteins away from this membrane. Thus, the accurate control of PM membrane turnover through regulation of transport to and endocytosis from the PM is crucial for its composition and function.

In the nervous systems of metazoans, stimulus detection occurs through sensory neurons, which are polarized cells with an apical PM whose composition is optimized for stimulus detection. Photoreceptors (PRs) are an excellent example of such sensory cell specialization. The apical PM of PRs is populated with molecules needed to transduce light, including the G-protein-coupled receptor (GPCR) rhodopsin, whose ability to absorb photons is fundamental to the initiation of phototransduction (Yau and Hardie, 2009). The importance of correct rhodopsin levels in PRs is exemplified by the condition night blindness, where dietary deficiency of vitamin A, the chromophore that binds rhodopsin, results in an inability to see in low light. Furthermore, genetic changes resulting in point mutations in rhodopsin lead to a reduction in the levels of this protein, which is associated with retinal degeneration; this is one of the major causes of inherited retinitis pigmentosa in humans (Hollingsworth and Gross, 2012). Thus, the accurate control of rhodopsin levels and localization is critical to normal PR structure and function.

In *Drosophila melanogaster*, vision is mediated by a compound eye composed of repeating units, ommatidia, that include eight sensory PRs, whose apical PM is expanded into microvilli to form the photosensitive membrane called the rhabdomere (Raghu et al., 2012). In these cells, sensory transduction is triggered when the GPCR rhodopsin 1 (Rh1 also known as NinaE) on the PM absorbs a photon of light that is transduced into electrical activity through G-protein-coupled phospholipase C (PLC) function (Hardie and Raghu, 2001). Rh1 is the most abundant protein on the PM, and its levels and localization are tightly controlled. Mutations that impair post-translational modification, folding and vesicular transport of Rh1 to the PM all reduce its levels at the rhabdomere (Baker et al., 1994; Li et al., 2007; Rosenbaum et al., 2006; Satoh et al., 2005). Rh1 levels at the rhabdomere are also regulated by light-activated endocytic turnover. Following photon absorption, Rh1 is phosphorylated, binds arrestin and is removed from the PM by CME to a Rab5-labelled compartment from where it is either recycled or degraded via late endocytic compartments (Xiong and Bellen, 2013). Defects at any of these steps result in Rh1 accumulation in endosomes (Alloway et al., 2000; Chinchore et al., 2009; Kiselev et al., 2000) and, in some cases, lead to retinal degeneration under illumination. Thus, the controlled biogenesis and transport of Rh1 during pupal development (pd), as well as its regulated endocytosis, are both essential to maintain PR structure and function.

Phosphoinositides are regulators of membrane turnover in eukaryotic cells (reviewed in Mayinger, 2012; Raiborg et al., 2016). For example, the lipid phosphatidylinositol 4-phosphate (PI4P) regulates exit from the Golgi (Hama et al., 1999), phosphatidylinositol 3-phosphate (PI3P) regulates early endosomal function (Stenmark et al., 1998), and the accurate control of phosphatidylinositol 4,5-bisphosphate [PI(4,5)P_2_] levels is critical for normal CME at the PM (Antonescu et al., 2011). Consequently, alterations in the levels of enzymes that generate or degrade PI(4,5)P_2_ results in profound defects in CME. The main route by which PI(4,5)P_2_ is synthesized is through phosphorylation of PI4P at position 5 by the activity of phosphatidylinositol 4-phosphate 5-kinase (PIP5K). Reduction in PIP5K activity causes a reduction in CME (Di Paolo et al., 2004), and mutants in synaptojanin and OCRL [PI(4,5)P_2_ 5-phosphatases] also impair CME (Erdmann et al., 2007; Mani et al., 2007).

PI(4,5)P_2_ can also be synthesized by a distinct class of enzyme, phosphatidylinositol 5-phosphate 4-kinases (PIP4K), which generate PI(4,5)P_2_ by phosphorylating phosphatidylinositol 5-phosphate (PI5P) at position 4 (Rameh et al., 1997). Owing to the low abundance of the substrate PI5P, PIP4K is not thought to regulate the main cellular pool of PI(4,5)P_2_, and knockdown of PIP4K in multiple systems is not associated with reductions in the levels of PI(4,5)P_2_ (Gupta et al., 2013; Jones et al., 2006; Weinkove et al., 2008). While the role of PIP5K in regulating CME is well established, to date, PIP4K enzymes have not been implicated in the regulation of endocytic pathways. In this study, we report that loss of PIP4K in *Drosophila* (Gupta et al., 2013) results in the redistribution of Rh1, a protein normally localized to the apical PM, to the cell body. This redistribution of Rh1 was associated with expansion of endomembrane compartments in PRs. These defects in Rh1 trafficking and endomembrane homeostasis could be rescued by downregulating CME but not by enhancing retromer-dependent recycling in the loss-of-function (protein null) mutant *PIP4K^29^*. Thus, we propose that PIP4K as a novel regulator of CME, controlling PM receptor composition and turnover during PR development in *Drosophila*.

## RESULTS

### Expanded endomembranes in *PIP4K^29^* PRs formed under illumination

**Fig. 1.**
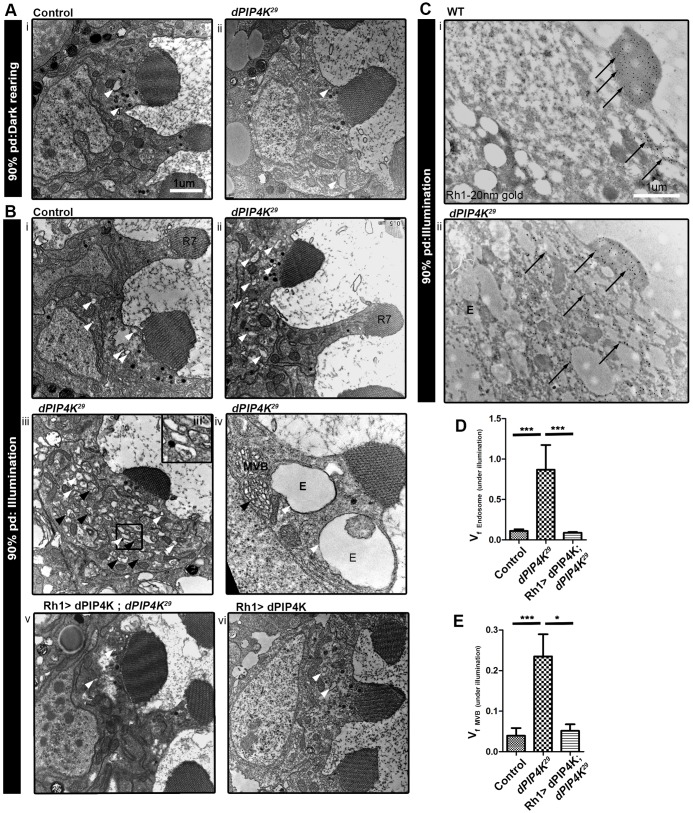
**Loss of PIP4K leads to the formation of expanded endomembranes under illumination.** (A,B) TEMs showing transverse sections of PRs from control (Ai) and *PIP4K^29^* (Aii) pupae during late (90%) pupal development (pd) reared in the dark; and control (Bi), *PIP4K^29^* (Bii–iv), Rh1>PIP4K; *PIP4K^29^* (Bv) Rh1>PIP4K PRs (Bvi) reared under illumination. The white arrowheads indicate endosome-like vesicles (labelled E) and black arrowheads indicate the MVBs, both of which are expanded in *PIP4K^29^* PRs under illumination (Bii–iv). R7, R7 PR cell. (C) Immuno-gold labelling for Rh1 in WT (i) and *PIP4K^29^* PRs (ii). Arrows point to immunogold particles labelling Rh1, which are mostly present in the rhabdomere or apical plasma membrane (APM) in WT and in cytosolic endosome-like vesicles in *PIP4K^29^* PRs. Scale bars: 1 μm. (D) Volume fraction analysis (VFA) for endosome-like vesicles in control, *PIP4K^29^* and Rh1>PIP4K; *PIP4K^29^* PRs under illumination at 90% pupal development. (E) VFA for MVBs under illumination at 90% pupal development. The values indicate the mean±s.e.m. of volume fraction in arbitrary units. *P<0.05; ***P<0.001 (one-way ANOVA with a nonparametric with Kruskal–Wallis test and Dunn's post test to compare the variance across samples).

While investigating PR ultrastructure in *PIP4K^29^* mutants, a loss-of-function mutant in *PIP4K* (denoted as *dPIP4K* in figures) (Gupta et al., 2013), we noticed defects in endomembrane homeostasis when flies were reared under illumination (wavelength of 400–700 nm; ∼3000 lux) during pupal development. Transmission electron microscopy (TEM) of retinae at 90% pupal development (when the biogenesis of apical PM is still in progress) from *PIP4K^29^* mutants reared under illumination revealed an increase in the number of vesicular endosome-like compartments (Fig. 1Bii,iii,iii′) as well as vesicles that were enlarged in size, in some cases as large as >1 µm^2^ (Fig. 1Biv). In addition, the cell bodies of *PIP4K^29^* PRs showed an expansion of multi-vesicular bodies (MVBs) (Fig. 1Biii,iv). Quantification of these structures by volume fraction analysis (VFA) revealed that these expanded endomembranes were rarely seen in controls reared under illumination (Fig. 1Bi,D,E) or in *PIP4K^29^* mutants reared in the dark (Fig. 1Aii). The expanded endomembranes in *PIP4K^29^* mutants could be rescued by reconstituting the animals with a wild-type (WT) *PIP4K* transgene (Fig. 1Bv,D,E). In WT PRs, Rh1 is restricted largely to the rhabdomere (Fig. 1Ci) whereas in *PIP4K^29^*, in addition to the rhabdomere, Rh1 was detected in the cell body on structures that resembled endosomes and MVBs (Fig. 1Cii; Fig. S1A). These results imply that, during pupal development under illumination, absence of PIP4K results in expansion of endomembrane compartments that contain the Rh1 in PRs.

### PIP4K supports Rh1 trafficking during pupal metamorphosis

We also found that when *PIP4K^29^* flies were reared under illumination during pupal development, the levels of Rh1 protein in freshly eclosed flies were reduced (Fig. S2A) compared to that seen in WT. This was associated with increased protein levels of the early endosome (EE) markers EEA1 (also known as Rabenosyn-5 in *Drosophila*) and Rab5 (Fig. S2B).

**Fig. 2.**
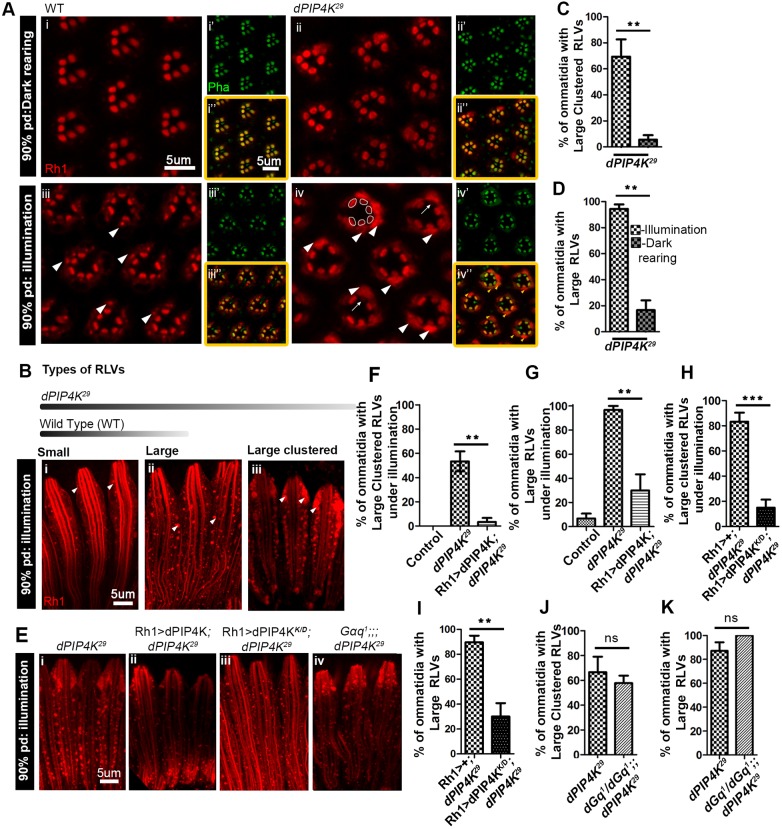
**Altered apical plasma membrane trafficking in *PIP4K^29^* PRs.** (A) Transverse sections through pupal retinae at 90% pupal development (pd), immunostained for Rh1 (red) and F-actin (green), for WT flies reared in the dark (i–i″) and under illumination (ii–ii″), and *PIP4K^29^* flies reared in dark (iii–iii″) and under illumination (iv–iv″). Arrowheads indicate the RLVs for WT (ii) and for *PIP4K^29^* flies, where the RLVs are highly expanded and form clusters filling up the cell bodies of PRs (iv). For reference, the rhabdomeres are outlined for 1 ommatidium. Arrows indicate missing Rh1 staining at the rhabdomere in some PRs that show RLV clusters. (B) Longitudinal sections of PRs at 90% pupal development, stained for Rh1 (red) for flies reared under illumination. Arrowheads indicate morphologically distinct types of RLVs – small RLVs, typical of WT (i), large RLVs (ii) and large clustered RLVs (iii) prevalent in *PIP4K^29^* (the bar at the top is a schematic showing what type of RLVs are represented in WT versus *PIP4K^29^*). (C,D) Quantification of the percentage of ommatidia containing large clustered RLVs and large RLVs in *PIP4K^29^* flies under illumination versus those kept in the dark at 90% pupal development. (E) Rh1 staining (red) in *PIP4K^29^* (i), Rh1>PIP4K;*PIP4K^29^* (ii), Rh1>PIP4K ^K/D^;*PIP4K^29^* (iii), *Gαq^1^*; *PIP4K^29^* (iv) PRs at 90% pupal development, from flies reared under illumination. (F,G) Quantification of large clustered RLVs and large RLVs, in *PIP4K^29^* flies reconstituted with Rh1>PIP4K versus *PIP4K^29^* flies, reared under illumination. (H,I) Quantification of large clustered and large RLVs, in *PIP4K^29^* flies reconstituted with Rh1>PIP4K ^K/D^ (kinase dead) versus *PIP4K^29^*, reared under illumination. (J,K) The percentage of ommatidia with large clustered RLVs and large RLVs in *Gq^1^*; *PIP4K^29^* versus *PIP4K^29^* are not different. **P<0.01; ***P<0.001; ns, not significant.

Since Rh1 levels were reduced at eclosion, we monitored Rh1 biogenesis and trafficking during development. We performed Rh1 immunolabelling in PRs at 78% pupal development [when Rh1 biogenesis and transport to the rhabdomere begins (Satoh and Ready, 2005)], 90% pupal development and in newly eclosed flies. At ∼78% pupal development, WT PRs reared under illumination showed partial staining of the expanding rhabdomere as well as punctate Rh1-loaded vesicles (RLVs), and a similar distribution of Rh1 was seen in *PIP4K^29^* mutants (Fig. S2C). However, by 90% pupal development, *PIP4K^29^* PRs showed extremely large clusters of RLVs (white arrowheads in Fig. 2Aiv,Biii) that were absent in WT (Fig. 2Aiii,Bi). Such RLV clusters in *PIP4K^29^* mutants were only common when the flies were reared under illumination, and only very rarely seen in dark-reared animals (Fig. 2Aii).

We classified Rh1 immunoreactive vesicles seen when reared under illumination in longitudinal sections (LS) into three categories, based on size and morphology (Fig. 2B): (1) small RLVs, which were the majority of RLVs in WT PRs; (2) large RLVs, which were observed commonly in *PIP4K^29^* PRs and very rarely in WT; and (3) large clustered RLVs, observed only in *PIP4K^29^* retinae. The latter two types could be quantified and showed a clear dependence on illumination in *PIP4K^29^* PRs (Fig. 2C,D). In *PIP4K^29^* mutants, the proportion of ommatidia with large clustered RLVs and large RLVs was much greater than in controls, and these phenotypes could be suppressed by reconstituting *PIP4K^29^* mutants with WT PIP4K protein (Fig. 2Eii,F,G). A kinase-dead transgene of PIP4K (Gupta et al., 2013) was also able to rescue this phenotype (Fig. 2Eiii,H,I). We tested the role of G-protein signalling in the light-dependent RLV clustering in *PIP4K^29^* mutants. The *Drosophila* *Gq^1^* (*Gq* is also known as *Gαq*) allele is a strong hypomorph for Gq function in PRs (Scott et al., 1995) and uncouples PLC signalling from photon absorption by Rh1. We generated *Gq^1^; PIP4K^29^* double mutants and found that, during illumination, the accumulation of RLVs was no different in *Gq^1^;PIP4K^29^* to that in *PIP4K^29^* (Fig. 2Eiv,J,K). This observation strongly suggests that ongoing Gq–PLC signalling is not required for the formation of RLV clusters in *PIP4K^29^*.

At eclosion, *PIP4K^29^* retinae grown under illumination had largely resolved these accumulations of large clustered RLVs, although there were clearly more residual large RLVs than in controls (Fig. S2D,E). A similar vesicular-trafficking defect was also seen for the Transient receptor potential (TRP) channel (Fig. S2F), an integral membrane protein of the apical PM, but was not observed for the Na-K ATPase, an integral protein of basolateral PM (Fig. S2G). These defects in Rh1 localization in *PIP4K^29^* mutants during late pupal development were not associated with defects in rhabdomere formation and structure since F-actin staining of the rhabdomere (Fig. 2Ai″–iv″) and rhabdomere ultrastructure as observed in TEM (Fig. S1Biv,v) were unaffected. These findings support the idea that PIP4K is required to support trafficking of integral apical PM proteins in PRs during illumination.

### An expanded early endosomal compartment underlies the Rh1-trafficking defects in *PIP4K^29^* mutants

We found no differences in the distribution of ER and Golgi markers between WT and *PIP4K^29^* PRs (Fig. S3A,B,C). The distribution of Myosin V (MyoV, also known as Didum), which is required for the transport of Rh1 from the Golgi to the rhabdomere (Li et al., 2007), at the rhabdomere terminal web (RTW) was unaltered in *PIP4K^29^* mutants (Fig. S3D). Furthermore, the distribution of Spam (also known as Eyes shut), a protein transported by a Golgi–exocytic route similar to that undertaken by Rh1 but localized to the intra-ommatidial space (Husain et al., 2006; Iwanami et al., 2016), was unaffected in *PIP4K^29^* mutants (Fig. S3E). In contrast to the light-dependent phenotype of *PIP4K^29^* mutants, post-Golgi exocytic transport of the Rh1 to the rhabdomere is believed to be independent of light (Li et al., 2007 and references therein). Collectively, these findings imply that defects in the transport of Rh1 to the apical PM, following synthesis in the ER and post-translational modifications at the Golgi, are unlikely to account for the endomembrane expansion and Rh1 mislocalization to vesicles in *PIP4K^29^* mutants.

**Fig. 3.**
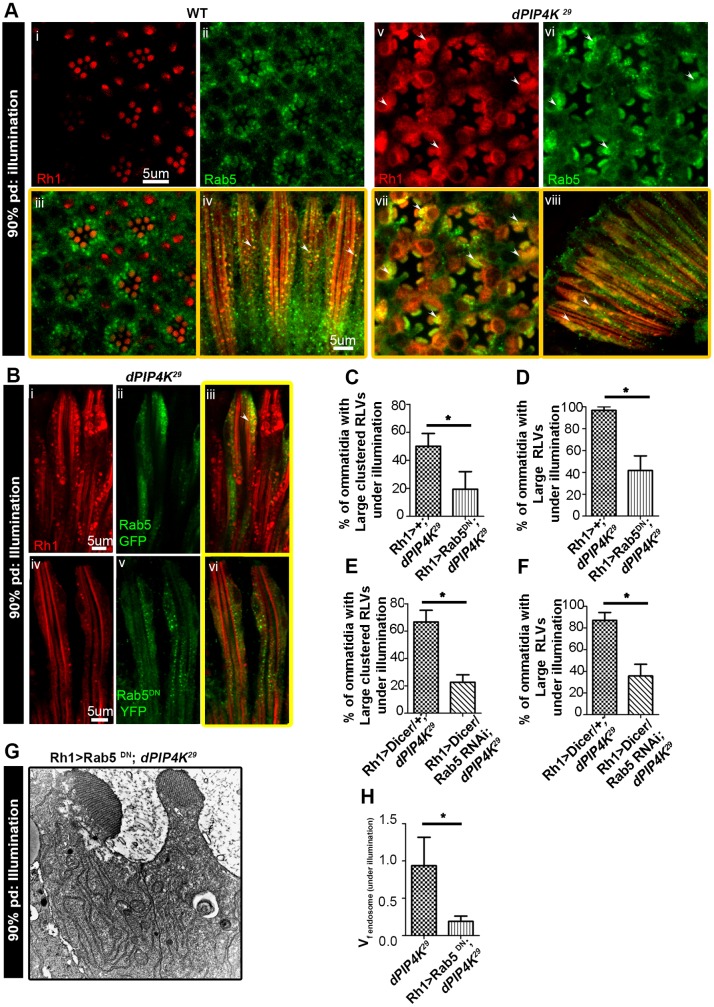
**Large RLV clusters in *PIP4K^29^* colocalize with early endosome markers.** (A) Double immunolabelling of WT (i–iv) and *PIP4K^29^* (v–viii) PRs for Rh1 (red) and Rab5 (green) at 90% pupal development (pd) for flies reared under illumination. In *PIP4K^29^* flies, the RLV clusters colocalize with Rab5, as indicated by arrowheads. (B) Rh1>GFP–Rab5 (green) also colocalizes with the large clustered RLVs (red) in *PIP4K^29^* flies (i), while Rh1>YFP–Rab5^DN^ YFP (green) in *PIP4K^29^* flies highlights fewer large clustered RLVs and is only rarely colocalized with RLVs (ii). (C–F) Quantification of large clustered RLVs and large RLVs in the lines Rh1>YFP-Rab5^DN^; *PIP4K^29^* versus *PIP4K^29^* and Rh1>Rab5 RNAi; *PIP4K^29^* versus *PIP4K^29^* (refer to Fig. 1Bii,iii,iv). (G) Electron micrographs show that there a lesser degree of expansion in endomembranes in the line Rh1>GFP-Rab5^DN^; *PIP4K^29^* with respect to that in *PIP4K^29^*. (H) VFA for endosome-like vesicles in Rh1>YFP-Rab5^DN^; *PIP4K^29^* versus *PIP4K^29^* for flies reared under illumination at 90% pupal development. *P<0.05.

By contrast, we found that during illumination, *PIP4K^29^* PRs contain an expanded Rab5-positive EE compartment (Fig. 3A). We found that the large clusters of Rh1 seen in *PIP4K^29^* colocalize with this expanded Rab5 compartment (Fig. 3Avii,viii) suggesting that, during illumination in the absence of PIP4K, Rh1 localizes to an expanded EE compartment.

We tested the effect of downregulating Rab5 function in *PIP4K^29^* mutants. Expression of GFP::Rab5WT in *PIP4K^29^* PRs had no consequence on Rh1 localization (Fig. 3Bi–iii); however, expression of dominant-negative Rab5 (Rab5^DN^::YFP) resulted in a significant reduction in the proportion of PRs with large and clustered RLVs (Fig. 3Biv–vi,C,D). TEM analysis also showed that the expansion of endomembranes seen in *PIP4K^29^* mutants was largely abolished following the expression of the Rab5^DN^::YFP (Fig. 3G,H). In addition, depletion of Rab5 through expression of a Rab5 RNAi driven by the *Rh1* promoter (denoted Rh1>Rab5^RNAi^) in *PIP4K^29^* flies resulted in a reduction in large and clustered RLVs (Fig. 3E,F). Collectively, these findings suggest that the expanded Rh1-containing endomembranes in the cell body of *PIP4K^29^* mutants are expanded EE Rab5-positive compartments.

### Normal endosomal maturation in *PIP4K^29^* mutants

**Fig. 4.**
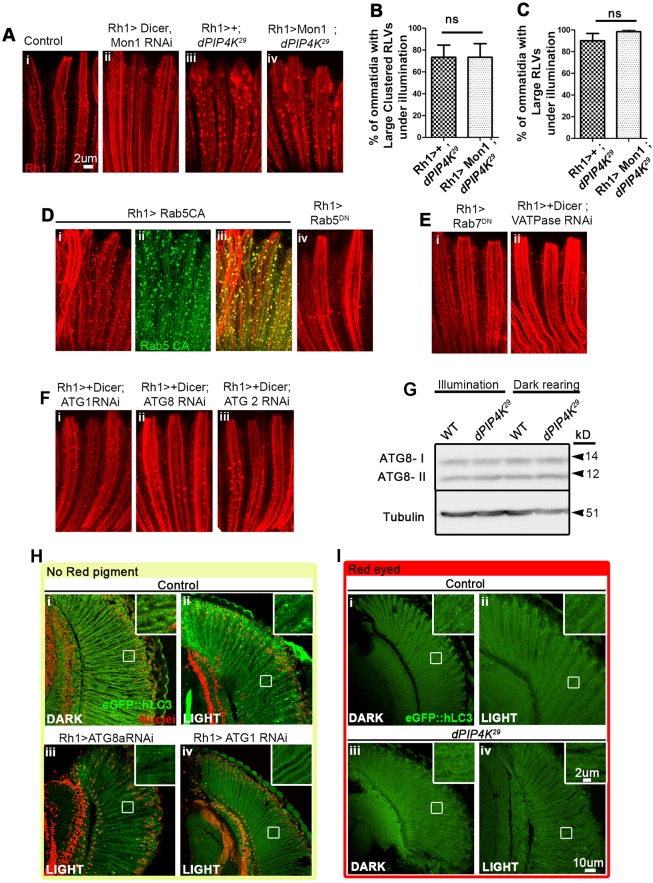
**Unaltered endo-lysosomal flux in *PIP4K^29^*.** (A) Rh1 immunostaining (red), in control (i) and Rh1>Mon1 RNAi lines (ii) showing an increase in the number of small RLVs with respect to the number seen in WT controls. RLVs stained in (iii) *PIP4K^29^* and (iv) Rh1>Mon1; *PIP4K^29^* are comparable. (B,C) Quantification of large clustered RLVs and large RLVs in Rh1>Mon1; *PIP4K^29^* versus *PIP4K^29^*. (D) Rh1>YFP-Rab5^CA^ (constitutively active) flies show an increase in the number of large RLVs (i–iii); these RLVs are not as expanded or clustered as is observed in the *PIP4K^29^* line. (iv) There are fewer RLVs (red) in Rh1>Rab5^DN^ than in WT. (E) (i) Rh1>Rab7^DN^ shows an increase in the number of small RLVs with respect to WT. (ii) Rh1>V-ATPase (Vha44) RNAi causes no alteration in RLVs. (F) Downregulating components of the canonical autophagy pathway [Rh1>ATG1 (i), ATG8 (ii) and ATG2 (iii)] causes no change in RLV organization. (G) Western blot analysis of autophagy. Head extracts from *PIP4K^29^* flies reared under illumination or in the dark showed no alteration in the autophagosome-associated, lipidated ATG8-II band with respect to that seen in WT reared under the same conditions. (H,I) Analysis of autophagy with the eGFP::hLC3 reporter. (H) Flies lacking the red (WT) screening pigment from the retinae, when exposed to constant illumination, showed accumulation of eGFP::hLC3 (green) in autophagosomes as punctae (ii and inset) whereas those reared in the dark did not, suggesting that autophagy is induced under illumination in the absence of screening pigment in the eye. ATG8 and ATG1 RNAi in the retinae under the similar conditions inhibit LC3 puncta formation (iii, iv). (I) WT flies (with the red screening pigment) showed no increase in eGFP::hLC3 puncta when reared under illumination compared to that seen upon dark rearing (ii versus i); *PIP4K^29^* mutants, which also have an equivalent red eye colour (screening pigment), showed no change in LC3 puncta under illumination, implying no change in autophagic flux in red-eyed flies under light versus dark rearing. ns, not significant.

An expanded EE Rab5 compartment and RLV clustering may arise from a block in the maturation of the EE Rab5 compartment to a late endosome (LE) compartment or through recycling mechanisms. To test this, we downregulated Mon1 in WT flies, because Mon1 is required for the replacement of Rab5 with Rab7 during the conversion of EEs into late endosomal vesicles (Poteryaev et al., 2010). In Rh1>Mon1^RNAi^, we found an increase in the number of RLVs, most likely due to an accumulation owing to a defect in maturation, but no evidence of large or clustered RLVs (Fig. 4Ai,ii). Furthermore, overexpression of Mon1 failed to rescue the RLV clustering seen in *PIP4K^29^* mutants (Fig. 4Aiii,iv,B,C), suggesting that reduced Mon1 function does not underlie RLV clustering.

We expressed Rh1>Rab7^DN^ (which blocks the replacement of Rab5 by Rab7) in WT PRs. This resulted in an increase in the number of RLVs, but failed to recapitulate the large and clustered RLVs seen in *PIP4K^29^* mutants (Fig. 4Ei). These observations suggest that a failure to convert the Rab5 compartment into a Rab7 compartment is unlikely to underpin the expanded Rab5-positive compartment seen in *PIP4K^29^* mutants. Consistent with this interpretation, we also found that large RLVs and large clustered RLVs in *PIP4K^29^* mutants partly colocalize with the late endosomal and lysosomal markers Rab7 (Fig. S3Fi–iii) and LAMP1 (Fig. S3Fiv–vi), respectively.

To test the contribution of defective late endosome–lysosome fusion in the formation of large clusters of RLVs, we downregulated the lysosomal proton pump vATPase (Rh1>dicer; vATPase *Vha44*^RNAi^) using a transgenic RNAi that has previously been shown to impact on lysosomal function (Mauvezin et al., 2015). This resulted in no detectable change in RLV numbers, and also did not result in RLV clustering in pupal PRs during illumination (Fig. 4Eii). This observation implies that defective lysosomal function is not sufficient to result in RLV cluster formation. Collectively, these observations imply that a defect in the maturation of the Rab5 endocytic compartment into late endosomes and in late endosome fusion with lysosomes is unlikely to lead to RLV clustering in *PIP4K^29^* mutants. Previous studies have suggested that the transport of Rh1 in late endocytic compartments is associated with its degradation (Chinchore et al., 2009). Our finding of Rh1 in Rab7- and LAMP1-positive compartments in *PIP4K^29^* suggests that the lysosomal degradation of Rh1 may lead to the reduction in Rh1 protein levels seen at eclosion (Fig. S2A).

### Defective autophagy does not underlie RLV clustering in *PIP4K^29^* mutants

It has recently been reported that a mammalian ortholog of PIP4K may regulate autophagy in human cell lines (Vicinanza et al., 2015). We tested the potential role of altered autophagy in the formation of large RLV clusters in *PIP4K^29^* mutants. In WT PRs downregulation of ATG1 (Fig. 4Fi), required for Target of rapamycin (TOR)-mediated initiation of autophagy or ATG8 (ATG8A), required for autophagophore expansion did not result in any defects in RLV numbers or RLV cluster formation (Fig. 4Fii). Furthermore, downregulation of ATG2, which is required for recycling of phagophore machinery (Nagy et al., 2014), did not have any impact on RLV numbers or clustering (Fig. 4Fiii).

We monitored ongoing autophagy in PRs by using two widely reported approaches (Mauvezin et al., 2014). First, we determined that the ratio of the levels of ATG8-I (the cytoplasmic form) to ATG8-II (the active lipidated autophagosome-associated form) protein was comparable between WT and *PIP4K^29^* in both dark-reared and illumination conditions (Fig. 4G); this means that there is normal induction and progression of autophagy. In the second approach, we made use of the eGFP::hLC3 reporter line (Rusten et al., 2004). In white-eyed flies, which experience very high levels of illumination (due to the absence of screening pigment), illumination results in the generation of large numbers of GFP-positive LC3 punctae indicating induction of autophagy (Fig. 4Hi,ii). The formation of such punctae can be blocked by the downregulation of ATG1 or ATG8, which are required for initiation of autophagosome formation (Fig. 4Hiii,iv). By contrast, in WT flies with red eye colour that results from the presence of screening pigment (where the microvillar PM sees lower light intensity), light does not induce LC3::GFP punctae formation (Fig. 4Ii,ii). In *PIP4K^29^* with an equivalent red eye colour, there was no evidence of increased numbers of LC3::GFP punctae in either dark- or light-reared conditions (Fig. 4Iiii,iv), thus implying that autophagy is not altered in these *PIP4K^29^* mutants. Thus, altered induction of autophagy is unlikely to underlie the large RLV clusters seen in the absence of PIP4K. Finally, although PIP4K has been reported to be linked to TOR activity, we found that downregulation of TOR in the outer PRs could not recapitulate the Rh1-trafficking defects seen in *PIP4K^29^* mutants (Fig. S3G,Hiii,iv), and the overexpression of Rheb, a direct activator of TOR could not rescue the Rh1-trafficking defects seen in this mutant (Fig. S3Hi,ii,I,J).

### Defective recycling does not underlie Rh1-trafficking defects in *PIP4K^29^* mutants

**Fig. 5.**
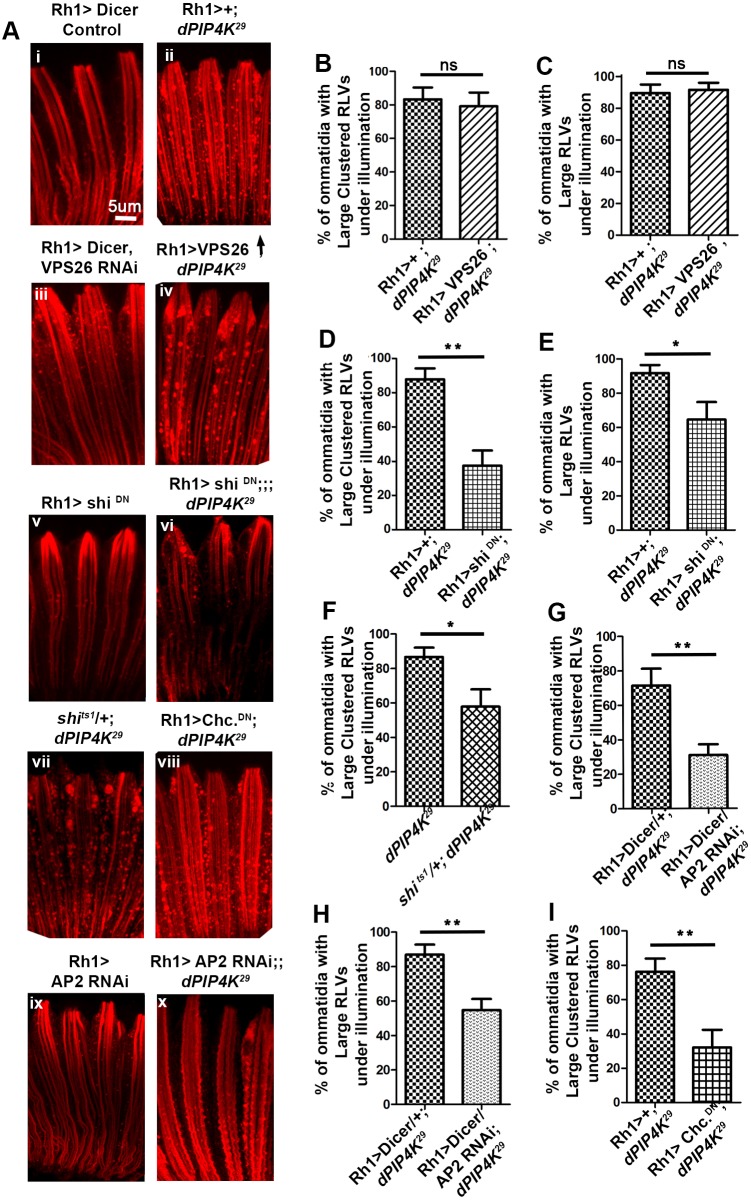
**Reducing CME rescues trafficking defects in *PIP4K^29^* PRs.** (A) Rh1 staining (red) in control (i), *PIP4K^29^* (ii), Rh1>Dicer vps26 RNAi (iii), Rh1>UAS-vps26; *PIP4K^29^* (iv), Rh1>shi^DN^ (v), Rh1>shi^DN^; *PIP4K^29^* (vi), *shi^ts1^*/+; *PIP4K^29^* (vii), and Rh1>Chc^DN^; *PIP4K^29^* (viii), Rh1>AP2 RNAi (xi) Rh1>AP2 RNAi; *PIP4K^29^* (x) PRs at 90% pupal development under illumination. (B,C) Quantification shows that there is no change in the percentage of ommatidia with large clustered RLVs and large RLVs in the Rh1>UAS-vps26; *PIP4K^29^* line with respect to that in *PIP4K^29^*. (D,E) A decrease in large clustered RLVs and large RLVs is observed in Rh1>shi DN; *PIP4K^29^* versus *PIP4K^29^* lines. (F,G) A decrease in large clustered RLVs is also observed in *shi^ts1^*/+; *PIP4K^29^* (F), and Rh1>Chc^DN^; *PIP4K^29^* lines with respect to that in *PIP4K^29^*. (H,I) A decrease in large clustered RLVs is also observed in Rh1>AP2 RNAi; *PIP4K^29^* lines versus *PIP4K^29^*. Thus, downregulating *Drosophila* CME partially suppresses the formation of large RLV clusters in *PIP4K^29^* flies reared under illumination. *P<0.05; **P<0.01; ns, not significant.

We also tested the role of defective recycling in the accumulation of RLVs in *PIP4K^29^* PRs. In *Drosophila* PRs, the recycling of endocytosed Rh1 depends on the activity of the retromer complex (Wang et al., 2014; Thakur et al., 2016). We tested the role of defective retromer function through downregulating Vps26, a core component of the retromer complex, by means of RNAi. In WT cells, downregulation of Vps26 (Rh1>dicer; Vps26^RNAi^) and Vps35 (data not shown) does not result in RLV cluster formation in PRs at 90% pupal development (Fig. 5Ai,iii). Furthermore, overexpression of Vps26 in *PIP4K^29^* PRs (Rh1>Vps26; *PIP4K^29^*), thus increasing retromer activity, was unable to rescue the RLV clustering (Fig. 5Aii,iv,B,C). Thus, reduced retromer-dependent recycling is unlikely to underlie the RLV clustering seen in *PIP4K^29^* mutants.

**Fig. 6.**
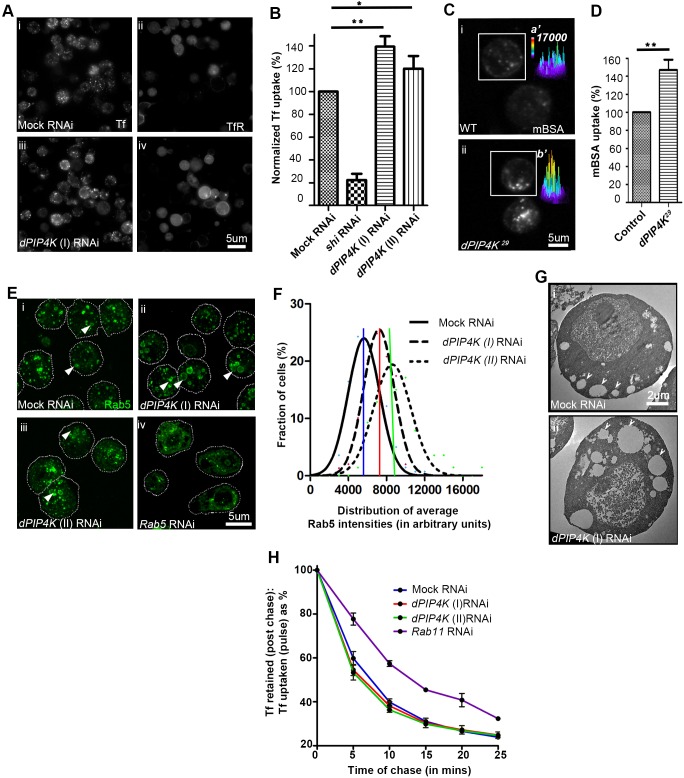
**Loss of PIP4K increases CME.** (A) S2R^+^ cells stably expressing human TfR were depleted of PIP4K (iii,iv) or mock (GFP) (i,ii) by using dsRNA, pulsed for 5 min with labelled Tf, to assay for CME, and the amount of uptake was normalized to the staining for surface TfR. (B) Quantitative analysis shows increased normalized Tf uptake in PIP4K knockdown cells versus mock-transfected cells. *P<0.05; **P<0.01 (C) *Drosophila* primary haemocytes showing labelled mBSA uptake and (D) quantification of same. **P<0.01 (one-way ANOVA with Dunnett's comparison of each column to control). (E) Rab5 staining (green) of cells expressing mock RNAi (i), and two different PIP4K dsRNAs (ii,iii). Arrowheads indicate enlarged Rab5 vesicles observed more frequently in PIP4K knockdowns. The cell outlines are indicated by dotted lines. (F) Quantification shows a right shift (increase), in the peak of population distribution (mode), for the mean Rab5 intensities per cell for PIP4K RNAi versus mock RNAi. (G) TEM images showing expanded endomembranes (marked by white arrowheads) in PIP4K knockdown (ii) versus mock RNAi (i). (H) Alexa Fluor 568-conjugated Tf was pulsed for 18 min; then cells were washed and imaged over time. The plot shows the fraction of Tf retained in cells at each time point as a percentage of that in the first image acquired after the pulse. The kinetics of Tf recycling was determined using a steady-state approach assay. The plot shows that the Tf is recycled to the cell surface in two phases – a fast decline phase and a slow phase. Knockdown of Rab11 slows the fast phase of Tf recycling with respect to mock RNAi. Knockdown of PIP4K using two different dsRNAs had no affects on the kinetics of Tf recycling.

To test the contribution of recycling pathways in the development of expanded endosomes during PIP4K depletion, we utilized *Drosophila* S2 cells stably expressing human transferrin (Tf) receptor (hTfR), where, following endocytosis, transferrin receptor recycling to the cell surface is mediated by a Rab11-dependent pathway. We performed a live-cell recycling assay, where following a saturating pulse of labelled human Tf (for 18 min) (Gupta et al., 2009), this cargo is recycled back to the cell surface with a *t*_1/2_ of ∼7 min (Fig. 6H). As a positive control, we depleted Rab11 (Xie et al., 2016) by means of RNAi, resulting in a slowing of recycling with *t*_1/2_ of ∼13 min (Fig. 6H). When PIP4K was depleted using two independent RNAi double-stranded RNAs (Fig. S4B), no significant change in the kinetics of Tf recycling was observed (Fig. 6H).

### Reducing CME rescues Rh1-trafficking defects in *PIP4K^29^* mutants

In WT flies, illumination increases Rh1 endocytosis into a Rab5-positive compartment (Han et al., 2007) via CME (Alloway et al., 2000; Kiselev et al., 2000). Since we found no defect in the maturation of the Rab5 compartment that might explain its expansion in *PIP4K^29^* mutants, we tested whether light-induced endocytosis of Rh1 through enhanced CME, might underlie the enlarged and clustered RLV formation in *PIP4K^29^* mutants. When dynamin function was partially suppressed [by manipulating *shibire* (*shi*), the *Drosophila* ortholog of dynamin], the accumulation of RLV clusters in *PIP4K^29^* during illumination was reduced (Fig. 5Av–vii,D–F). Likewise, reduction in clathrin adaptor protein 2 (AP-2) function through expression of α-adaptin RNAi (Rh1>α-adaptin^RNAi^) (Fig. 5Aix,x,G,H) or a clathrin dominant-negative construct (Rh1>*chc*^DN^) in *PIP4K^29^* mutants (Fig. 5Aviii,I) resulted in a reduction in RLV clustering during illumination. These observations strongly suggest that, during illumination in *PIP4K^29^* mutants, enhanced CME could contribute to Rh1 accumulation in an expanded Rab5 compartment.

### PIP4K is a negative regulator of CME

We monitored Tf (a canonical cargo endocytosed by CME) uptake following RNAi-mediated depletion of PIP4K in *Drosophila* S2 cells stably expressing a human Tf receptor (Gupta et al., 2009) (Fig. 6A). When PIP4K was depleted with two separate RNAi sequences (Fig. S4B,C), the normalized (with respect to the surface Tf receptor level) uptake of Tf was enhanced even within a timescale of 5 min (Fig. 6A,B; Fig. S4A). Similar observations were also seen with 2 min uptake studies (data not shown), a time window where Tf has not likely entered recycling routes. Furthermore, in S2 cells depleted of PIP4K, the EE compartment marked by Rab5 (Fig. 6E,F) and EEA1/Rbsn-5 (Fig. S4D) was expanded; this finding is reminiscent of our observation of an expanded Rab5 compartment in *PIP4K^29^* PRs during illumination (Fig. 3A). In electron micrographs of S2 cells where PIP4K had been depleted, we observed increased large endomembrane compartments that were close to the plasma membrane compared to the smaller vesicles in mock-treated cells (Fig. 6G). We also found that uptake of malelyated bovine serum albumin (mBSA) by larval haemocytes, a process known to depend upon CME (Abrams et al., 1992) was enhanced in *PIP4K^29^* compared to WT cells (Fig. 6C,D).

### PIP4K localizes to an early endosomal compartment

**Fig. 7.**
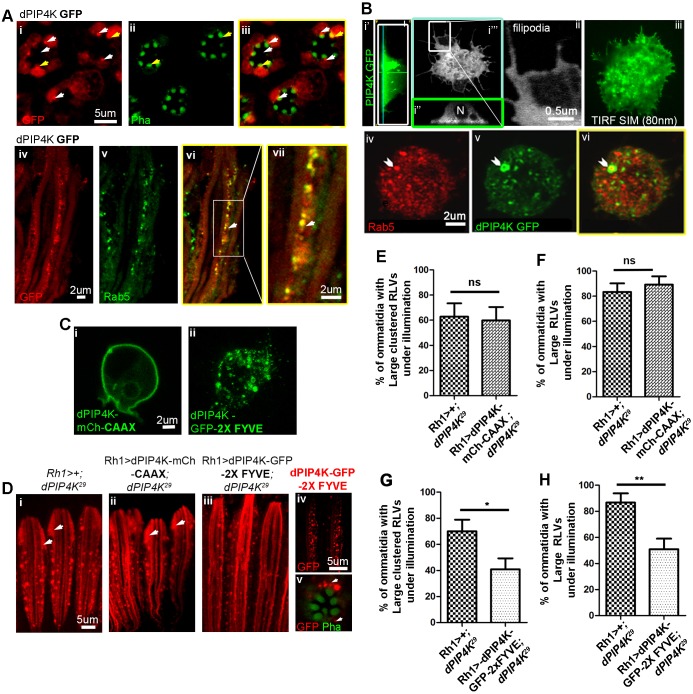
**PIP4K::GFP localizes to and is required in early endosomal compartments during CME.** (A) (i-iii) Transverse sections of PRs at 90% pupal development from Rh1>PIP4K::GFP flies reared under illumination. PIP4K::GFP partly localizes to the rhabdomere (APM) (stained with phalloidin, green) as indicated by the white arrows. PIP4K::GFP also localizes to the cell body of the PRs and occasionally is visible as distinct punctae close to the base of rhabdomere. (iv–vii) Longitudinal sections of ommatidia, showing the PIP4K::GFP (green) punctae colocalizing with early endosomal Rab5 (red) compartments. (B) Localization of PIP4K::GFP in S2R^+^ cells. (Bi′,i″) Longitudinal sections of S2R^+^ cells transiently expressing PIP4K::GFP, showing it to be broadly distributed, enriched close to PM near the coverslip and excluded from the nucleus (N). (Bi′″,ii) Confocal section showing that PIP4K::GFP is localized close to plasma membrane, and is occasionally visible as punctae and in the filopodial extensions. (Biii) TIFR-SIM image showing PIP4K localizing close to the PM (80 nm TIRF field). (Biv–vi) Occasionally PIP4K::GFP was present next to early endosome compartments marked by Rab5 (red) as highlighted by the chevrons. (C) Colocalization of CAAX-tagged PIP4K (dPIP4K-mCh-CAAX) (i) and 2×FYVE-tagged PIP4K::GFP (dPIP4K-GFP-2X FYVE) (ii) verified in S2 cells. (D) RLVs (red) in Rh1>+; *PIP4K^29^* (i), Rh1>PIP4K-mCherry-CAAX; *PIP4K^29^* (ii) and Rh1>PIP4K::GFP-2X FYVE; *PIP4K^29^* (iii) lines. Localization of PIP4K::GFP–2XFYVE in longitudinal sections (iv) and transverse sections (v) of PRs as distinct puncta or vesicles. (E–H) Quantification of the percentage of ommatidia containing large clustered RLVs and large RLVs in Rh1>PIP4K-mCherry-CAAX; *PIP4K^29^* and Rh1>PIP4K::GFP-2X FYVE; *PIP4K^29^* lines with respect to that in *PIP4K^29^*. PIP4K::GFP–2XFYVE partially suppresses formation of large clustered RLVs and large RLVs in *PIP4K^29^* flies. *P<0.05; **P<0.01; ns, not significant.

Previous biochemical studies have suggested that PIP4K co-fractionates with markers of the PM, Golgi and endolysosomes (Gupta et al., 2013). We studied the distribution of PIP4K::GFP in pupal PRs; under illumination, a fraction of the GFP-tagged protein localizes to the rhabdomere (yellow arrows Fig. 7Ai–iii) and partial localization to intracellular punctae close to the base of the rhabdomere is also seen (white arrows Fig. 7Ai–iii). Similar localization of PIP4K::GFP was seen when reconstituted in *PIP4K^29^* mutant PRs (data not shown). These PIP4K::GFP puncta partly colocalize with a Rab5-positive compartments (Fig. 7Aiv–vii). Importantly, this localization was seen when the reconstituted PIP4K::GFP protein was expressed at levels lower than that of endogenous PIP4K (Fig. S4F), suggesting that this localization is not an artefact of protein overexpression.

We also studied the localization of PIP4K::GFP when transiently expressed in *Drosophila* S2 cells. In 3D reconstructions of single cells (Fig. 7Bi), we found that PIP4K::GFP was broadly distributed (although excluded from the nucleus) (Fig. 7Bi″) and concentrated in regions close to the plasma membrane. It was also found in filopodia, which are extensions of the PM (Fig. 7Bi″′,ii). We also examined the distribution of PIP4K::GFP by using super-resolution structured illumination microscopy coupled with total internal reflection fluorescence (TIRF-SIM), which has a resolution of ∼80 nm. Under these conditions, we found PIP4K::GFP in the TIRF-SIM field, i.e. within 80 nm of the coverslip and thus extremely close to the PM (Fig. 7Biii). We also found that, in a few instances, PIP4K::GFP was juxtaposed next to endogenous Rab5 on vesicular structures (Fig. 7Biv–vi). Thus, in S2 cells, PIP4K::GFP can be detected close to the PM and partly overlapping with the Rab5 compartment.

### Reconstitution of PIP4K in early endosomes can rescue trafficking defects in *PIP4K^29^* mutants

To understand where the PIP4K that supports endocytic trafficking during CME is located, we reconstituted *PIP4K^29^* flies with PIP4K specifically targeted to the plasma membrane and the EE compartment. In order to selectively localize PIP4K to the plasma membrane, we added a CAAX motif (van Rheenen et al., 2007) (PIP4K::mCherry-CAAX), which restricts protein localization to the PM (Fig. 7Ci). When reconstituted into *PIP4K^29^* PRs, this protein was not able to rescue the accumulation of large RLVs and large clustered RLVs (Fig. 7Di,ii,E,F).

We also targeted PIP4K::GFP to the EE compartment by using the FYVE domain of EEA1 (Stenmark et al., 1998) (PIP4K::GFP–2×FYVE), which restricts the protein to a punctate vesicular compartment that likely represents PI3P-enriched vesicles (Fig. 7Cii,Div,v). We found that PIP4K::GFP–2×FYVE was able to achieve a substantial rescue in accumulation of large RLVs and large clustered RLVs in PRs (Fig. 7Diii,G,H), suggesting that PIP4K function is required in an EE compartment.

## DISCUSSION

During the development of polarized cells, apical domain-specific proteins need to be transported to and maintained at the apical plasma membrane. Such protein localization to the apical domain needs to be achieved in the face of endocytic turnover of the plasma membrane that is an ongoing process in cells. For example, during *Drosophila* PR development, the apical plasma membrane expands during late pupal development and is populated with phototransduction-specific molecules such as Rh1. At the same time, Rh1 at the plasma membrane is subject to ongoing clathrin-dependent endocytic turnover, a process that is essential for normal apical domain development (Raghu et al., 2009). The mechanism by which apical domain proteins such as Rh1 are retained or recycled to the plasma membrane following endocytosis has remained unclear.

In this study, we found that, in *Drosophila*, loss of the only PIP4K results in the abnormal expansion of endomembranes in the PR cell body. In *PIP4K^29^* cells the GPCR Rh1, which is endocytosed by CME, was found on these expanded endomembranes in contrast to what is seen in WT flies, where Rh1 is mainly localized to the apical plasma membrane. The Rh1 in the cell body of *PIP4K^29^* mutants colocalized with multiple endocytic compartments. These phenotypes could be reverted by downregulation of Rab5 activity, suggesting that a Rab5-dependent expansion of endomembranes underlies the Rh1-trafficking defect in cells depleted of PIP4K. A conceptually similar observation was seen in *Drosophila* S2 cells expressing the Tf receptor, which is also endocytosed by CME. In S2 cells depleted of PIP4K, the Rab5- and EEA1/Rbsn-5-positive endocytic compartment was expanded. Thus, the loss of PIP4K results in increased uptake of CME-dependent cargoes and an expansion in the size of the endolysosomal system.

In principle, an increase in the size of the Rab5-positive EE compartment can arise from the increased delivery of vesicles to it or from a failure of Rab5-positive endocytic vesicles to undergo maturation into downstream elements of the endosomal system (e.g. Rab7 compartment, fusion with lysosomes or maturation into recycling endosomes). We found that in WT PRs, blocking the maturation of the Rab5 compartment to a Rab7 compartment or inhibiting the fusion of Rab7 with lysosomes failed to recapitulate the Rh1-trafficking defects seen when PIP4K was absent. Thus, a defect in the maturation of the Rab5 compartment along the late endosome–lysosome system does not underlie the Rh1-trafficking defects seen when PIP4K function is depleted. Likewise, downregulating retromer function in PRs failed to recapitulate the phenotype of *PIP4K^29^* mutants, and enhancing retromer function, which was previously shown to clear RLVs from the cell body (Thakur et al., 2016; Wang et al., 2014), failed to rescue the Rh1-trafficking defect in *PIP4K^29^* mutants. Thus, the failure of retromer-dependent recycling is unlikely to explain the Rh1-trafficking defect in *PIP4K^29^* mutants.

By contrast, the expanded Rab5-positive compartment in *PIP4K^29^* mutants, which colocalizes with RLVs and large clustered RLVs, could be rescued by reducing CME at the plasma membrane suggesting that excessive endocytosis of Rh1 into RLVs underlies the phenotypes seen in these flies. Consistent with this model, we found that Tf uptake in S2 cells and the mBSA uptake in haemocytes, both of which depend on CME (Abrams et al., 1992; Gupta et al., 2009) was enhanced in cells depleted of PIP4K. Thus, it is likely that the expanded Rab5 compartment seen in cells depleted of PIP4K is underpinned by enhanced ongoing CME. Interestingly, this enhanced uptake seems specific for CME since the uptake of dextran by S2 cells, which is not mediated by CME, was not increased in PIP4K-depleted cells (data not shown).

**Fig. 8.**
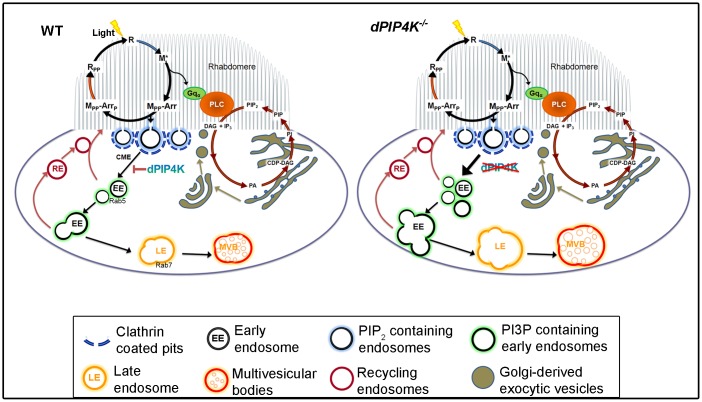
**Proposed role of PIP4K in regulating endocytic turnover of Rh1.** Rhodopsin (R) is activated by light to metarhodopsin (M*), which triggers GPCR activation and, in turn, causes PLC-mediated PIP_2_ turnover in the rhabdomere/apical plasma membrane of fly PRs. M* is bound by arrestin proteins (Arr) and endocytosed by CME. PIP4K regulates CME from the plasma membrane, and its absence causes increased CME uptake, leading to an increased number of Rhodopsin-loaded early endocytic vesicles (RLVs) that undergo homotypic fusion to form larger early endocytic compartments, and later mature normally as enlarged late endosomes and MVBs. RE, recycling endosomes.

This model of PIP4K function (Fig. 8) predicts that this protein might be localized either at or close to the plasma membrane. Through high-resolution imaging, we found that in both PRs and S2 cells, PIP4K was present very close to the plasma membrane with a proportion colocalizing with Rab5-positive structures. We found that reconstituting *PIP4K^29^* flies with PIP4K selectively targeted to the PI3P-enriched EE compartment (PIP4K::GFP–2×FYVE) resulted in a substantial (>50%) rescue of the RLV-clustering phenotype; this strongly suggests that PIP4K function is required in this compartment to regulate CME. The inability to fully rescue the Rh1-trafficking defect may reflect the requirement of PIP4K function in an additional non-PI3P enriched compartment. By contrast, we found that reconstituting *PIP4K^29^* cells with PIP4K function selectively at the PM (PIP4K::mCherry–CAAX) failed to rescue the Rh1-trafficking defect. This observation implies that PIP4K function at the PM is not sufficient to regulate Rh1 trafficking in PRs, and rather indicates a requirement of PIP4K function in the downstream PI3P-enriched EE compartments.

During CME, early events leading to clathrin-coated pit formation, a process dependent on accurate PI(4,5)P_2_ turnover (Antonescu et al., 2011; Nández et al., 2014), are followed by the biogenesis of early endosomes, a Rab5- and PI3P-dependent process (Zoncu et al., 2007). It has also been reported that enhancing Rab5 activity increases Tf receptor endocytosis (Bucci et al., 1994) and results in the formation of large early endosomes by homotypic fusion in mammalian cells (Stenmark et al., 1994). This is reminiscent of our observation where PIP4K depletion in S2 cells results in both enhanced Tf uptake and the enlarged Rab5-positive structures (Fig. 6A,B,E,F). Together with our observation that the Rh1 accumulation in *PIP4K^29^* PRs could be prevented by downregulating Rab5 function (Fig. 3), these observations imply that Rab5 activity might be enhanced by loss of PIP4K. Interestingly, while expression of a constitutively active Rab5 in WT PRs resulted in a clear increase in the number of large RLVs (Fig. 4Di–iii), these RLVs did not undergo further fusion to form large clustered RLVs and this may imply that additional PIP4K events, apart from Rab5 activity are required for RLV clustering in PRs. Although Rab5 localization has been reported at multiple early endocytic compartments, Zoncu et al. have reported that the Rab5-dependent enrichment of PI3P on early endosomes occur at a later time point, after the 5-phosphatase-dependent turnover of PI(4,5)P_2_ has been completed (Zoncu et al., 2007). The observation that reconstituting PIP4K in PI3P-enriched endosomes was sufficient to partly rescue the formation of RLV clusters (Fig. 4Dii,G,H) suggests, that the PIP4K-dependent regulation of Rab5 activity must occur once EEs have accumulated PI3P.

The Rh1-trafficking defect in *PIP4K^29^* PRs is only seen when these cells are exposed to light, triggering Rh1 isomerization and plasma membrane turnover. What is the mechanism underlying the light-dependent Rh1-trafficking defects in cells depleted of PIP4K? In this study, we found that a strong hypomorph in Gq was unable to suppress the Rh1-trafficking defect in *PIP4K^29^* mutants. This finding suggests that, although the RLV trafficking phenotype is light dependent, it does not require G-protein-coupled PLC signalling. This conclusion is consistent with our findings showing that basal levels of PI(4,5)P_2_ at the plasma membrane of *PIP4K^29^* PRs are normal (Fig. S4H), that light-induced PI(4,5)P_2_ turnover is not affected (Fig. S4G) and that the electrical response to light, a direct output of G-protein-coupled PLC signalling, is unaffected in *PIP4K^29^* mutants (Chakrabarti et al., 2015). The only known molecular event triggered by photon absorption prior to Gq activation is the photoisomerization of Rh1 (receptor ligand interaction) to form M*. M* can absorb a second photon, and undergo phosphorylation, arrestin binding and endocytosis (Raghu et al., 2012); it is likely that a PIP4K-dependent step in part of this endocytic process, thus, underlies the light-dependent Rh1-trafficking defects seen in *PIP4K^29^* mutants. This conclusion is consistent with our finding that downregulation of CME by using multiple methods was able to suppress the RLV cluster formation seen in *PIP4K^29^* mutants during illumination.

How does PIP4K regulate early endosomal dynamics during CME? One possibility that arises is the reduced TORC1 activity reported in *PIP4K^29^* mutants (Gupta et al., 2013) given the reported regulation of TORC1 by Rab5 (Bridges et al., 2012; Gli et al., 2010). However, we found that the downregulation of TORC1 in WT cells was unable to recapitulate the RLV clustering seen in *PIP4K^29^*. Thus, the reduced TORC1 activity reported on loss of PIP4K is insufficient to explain the RLV clustering. It is possible that other PIP4K-dependent mechanisms in EEs may regulate early endosomal dynamics. PI5P has been reported in EE compartments (Ramel et al., 2011). However, in view of the ability of a kinase-dead PIP4K to rescue early endosome expansion in *PIP4K^29^* mutants, it is likely that the conversion of PI5P to PI(4,5)P_2_ by PIP4K does not underlie this phenotype. Kinase-dead PIP4K could bind to and sequester PI5P or regulate the activity of other proteins that participate in CME. The relevant mechanism remains to be discovered.

In summary, because we have identified a role for PIP4K in regulating early endosomal dynamics during CME of multiple cargoes in several cell types, it is likely that this protein regulates some aspect of the core machinery of EE homeostasis during CME; the identity of the specific step remains to be established.

## MATERIALS AND METHODS

### Fly stocks and rearing conditions

All flies (*Drosophila melanogaster*) were reared on standard corn meal agar with 1.5% yeast, in 25°C incubators with no internal illumination, referred to as dark rearing. Wild type (WT) used were W^+^ Oregon-­R, the closest match to the eye pigmentation of the strains used in experiments. The GAL4–UAS system was used to express transgenic constructs. Rh1-GAL4 drives GAL4 expression in the peripheral PRs after 68% pupal development. For experimental purposes, a description stating that flies are reared under illumination refers to animals grown under constant light of wavelength 400–700 nm and intensity of ∼3000 lux throughout development.

The following fly lines were obtained from the Bloomington stock centre: *Gαq^1^* (B#42257), UAS-YFP Rab5.S43N (B#9771), UAS-GFP-Rab5 (B#43336), UAS-YFP.Rab5.Q88L (B#9774), UAS-Rab7.GFP (B#42705), UAS-YFP.Rab7.T22N (B#9778), UAS-ATG1 RNAi (B#35177), UAS-ATG2 RNAi (B#34719) and UAS-Chc^DN^ (B#26821). The following fly lines were obtained from the Vienna *Drosophila* resource centre: UAS-Rab5 RNAi(V#103945), UAS-AP2 RNAi(V#15565), UAS-Mon1 RNAi(V#38600), UAS-ATG8 RNAi(V#102155) and UAS-vATPase/vha 44 RNAi(V#101527). UAS-*shi*^DN^ and *shi^ts1^* were from Mani Ramaswami (Trinity College, Dublin, Ireland); UAS-VPS26-HA was from Hugo Bellen (Baylor College of Medicine, Houston, TX); UAS-vps26 RNAi and UAS-vps35 RNAi were from Miklós Sass (Eötvös Loránd University, Budapest, Hungary), and UAS-mtm::GFP was from Amy Kiger (Biological Sciences, UCSD, CA).

### Generation of transgenic lines

PIP4K cDNA was amplified along with the GFP tag at its C-terminus, from the pUAST-PIP4K::eGFP vector (Gupta et al., 2013). The FYVE domain DNA was amplified from genomic DNA of UAS-GFP-myc-2×FYVE flies (Amy Kiger). mCherry CAAX was amplified from pcDNA3-mCherry CAAX. Overlaps to be used for GIBSON assembly were introduced into all these inserts during the PCR step. The tandem 2×FYVE domain construct was made by sequential insertion of FYVE domains at the C-terminus of PIP4K–GFP in pUAST attB. The mCherry–CAAX sequence was inserted at the C-terminal of PIP4K after a flexible linker sequence (GGSGGGSGGGSG).

### Western blotting

Retinal extraction, sample preparation and western blotting were performed as per the protocol in Chakrabarti et al., (2015). Primary antibodies used were against: Rh1, 1:1000 (4C5, DSHB); tubulin, 1:4000 (E7, DSHB); Rab5, 1:1000; GM130, 1:1000 (Abcam 30637); PIP4K, 1:1000 (Gupta et al., 2013), EEA1 (Mario Zerial, MPI-CBG, Dresden, Germany) and *Drosophila* ATG8a, 1:1000 (Rachel Kraut, Biological Sciences, Nanyang Technological University, Singapore). All horseradish peroxidase (HRP)-coupled secondary antibodies (JIR) were used at 1:10,000 dilution.

### Immunohistochemistry

Dissection, fixation and staining method for PRs is described in Chakrabarti et al., (2015). Antibodies used were against: Rh1, 1:50 (4C5-C); Na-K-ATPase, 1:50 (a5-C); and Spam/Eys, 1:50 (21A6) (all DHSB); Rab5, 1:50 (Marcos Gonzalez-Gaitan, University of Geneva, Switzerland); GFP, 1:2000; and GM130, 1:400 (Abcam 30637); MyoV, 1:1000 (Donald F. Ready, Purdue University - Biology Department, Lafayette, IN) and hTFr, 1:50 (OKT9, Satyajit Mayor, NCBS, Bangalore, India). The incubation was performed overnight at 4°C. Secondary antibodies were used at 1:300 dilution [against IgG and conjugated to Alexa Fluor 633 or 568 (Molecular Probes)]. The whole-mounted preparations were imaged under 40× or 60×1.4 NA objective, in Olympus FV1000 microscope.

### Electron microscopy

Samples were prepared as described in Thakur et al. (2016). Ultrathin sections of ≤40 nm thickness were obtained on a Tecnai ultramicrotome and imaged on a Tecnai G2 Spirit Bio-TWIN (FEI) 120 kV electron microscope.

### Volume fraction analysis

The relative abundance of endosome-like vesicles and multivesicular bodies (MVBs) were estimated by volume fraction analysis (VFA) by a systematic point count method. A point grid (3000×3000 points over 10 μm^2^ area) was overlaid on the transverse section images of an ommatidium using the grid plugin in Fiji. The number of points/intersections of the grid falling on a structure of interest (e.g. endosome, or MVBs; P_E_ or P_MVB_, respectively) and within the reference space (in this case the apical PM or rhabdomere; P_ref_) were counted. VFA was quantified as: Vf_Endosome_=P_E/_P_ref_ or Vf_MVBs_=P_MVBs/_P_ref_.

The graphs indicate the mean+s.e.m. of volume fraction in arbitrary units from the six peripheral PRs across six ommatidia from six different flies in two independent experiments.

### Immunoelectron microscopy

Dissected retinae were fixed in 4% formaldehyde and 0.4% glutaraldehyde in 0.1 M PIPES for 30 mins at 4°C. Tissues were washed with 0.1 M PIPES and dehydrated in an ethanol series. The tissues were infiltrated with LR White resin series (Fluka) in 80% ethanol for 2 h at room temperature; tissue blocks were polymerized as per the manufacturer's protocol. Retinal cross sections of ∼50 nm thickness were cut and mounted on nickel grids (EMS). These sections were treated with 0.1% NaBH_4_ solution, after which they were incubated overnight in 1:25 dilution of anti-Rh1 antibody (4C5). Grids were washed and incubated with 1:50 gold (20 nm)-conjugated anti-mouse-IgG secondary antibody (EMS) for 2 h at room temperature. Grids were counterstained with 5% uranyl acetate and imaged on Tecnai G2 Spirit Bio-TWIN (FEI) electron microscope.

### Cell culture

S2R^+^ cells were cultured as described in Gupta et al. (2009) and primary haemocytes isolated from third-instar *Drosophila* larvae as described in Sriram et al. (2003).

### dsRNA-mediated RNA depletion

Primers containing 5′ T7 RNA polymerase-binding site (5′-TAATACGACTCACTATAGGG-3′) followed by a sequence specifically against the coding sequence of PIP4K were designed. dsRNA production, purification and transfection into S2 cells were performed as described in Worby et al. (2001). After this, the cells were replated, treated with a second round of 4 μg of dsRNA and cultured for 2 more days to allow turnover of target proteins.

### Endocytic uptake and recycling assays

Transferrin (Tf) and mBSA uptake assays were performed as previously reported (Gupta et al., 2009). The Tf recycling assay was performed on live S2R^+^ cells, in an approach to steady-state assay (Gupta et al., 2009, Johnson et al., 1993) where a saturating pulse of Tf was chased and imaged over time.

### TIRF-SIM imaging

2D-TIFR-SIM images were acquired on a Nikon N-SIM system using an Eclipse Ti microscope with a 100× objective and a 100 EX V-R diffraction grating block.

### Quantification of types of RLVs

Three different types of RLVs are defined based on size and morphology: (1) small RLVs, (2) large RLVs and (3) large clustered RLVs. In all quantitative analysis of the types of RLVs, the values represent the mean±s.e.m. percentage of ommatidia showing a particular class of RLVs, measured from ≥30 ommatidia from at least five different flies.

### Statistics

Unless otherwise indicated, *P*-values were determined by performing an unpaired nonparametric two-tailed *t*-test (Mann–Whitney), with *P<0.05; **P<0.01; ***P<0.001.
